# Bibliometric Trends in the Integration of Computer Vision With Healthcare

**DOI:** 10.1049/htl2.70085

**Published:** 2026-05-14

**Authors:** Rakhi Issrani, Hafiz Muhammad Zeeshan, Abid Iqbal, Shahzad Ahmad, Shazia Iqbal, Namdeo Prabhu, Muhammad Nadeem Baig

**Affiliations:** ^1^ Department of Research Analytics Saveetha Dental College and Hospitals Saveetha Institute of Medical and Technical Sciences Saveetha University Chennai India; ^2^ Department of Computer Science National College of Business Administration and Economics Lahore Pakistan; ^3^ Department of Computer Science Superior University Lahore Pakistan; ^4^ Central Library Prince Sultan University Riyadh Kingdom of Saudi Arabia; ^5^ Faculty of Medicine and Health Sciences The University of Buckingham Buckingham UK; ^6^ IMBB the University of Lahore Lahore Pakistan; ^7^ Department of Oral and Maxillofacial Surgery & Diagnostic Sciences College of Dentistry Jouf University Sakaka Kingdom of Saudi Arabia; ^8^ Department of Preventive Dentistry College of Dentistry Jouf University Sakaka Kingdom of Saudi Arabia

**Keywords:** artificial intelligence, computer vision systems, deep learning, healthcare

## Abstract

Computer vision, enabled by machine learning and image processing techniques, plays a crucial role in healthcare by facilitating the analysis of medical images such as magnetic resonance imaging, computed tomography and X‐rays. It supports disease diagnosis, treatment planning, telemedicine and remote patient monitoring. Despite rapid growth, research in this field remains fragmented, making it difficult to comprehensively understand its evolution and research patterns. This study aimed to systematically analyse global research trends in computer vision applications in healthcare using bibliometric methods. A bibliometric analysis was conducted on 1023 publications retrieved from the Web of Science (Wos) database published between 2000 and 2022. Scientific output, citation patterns, leading journals, authors, institutions, countries, funding agencies and research themes were analysed using VOSviewer, Biblioshiny and ScientoPy. The annual scientific output showed a consistent upward trend, with a peak in 2021. IEEE Access emerged as the most productive journal (90 publications), while IEEE transactions on medical imaging was the most cited source. China led in publication output and international collaboration, followed by the United States. Keyword analysis revealed dominant themes such as deep learning, image segmentation, classification and medical imaging. This bibliometric analysis provides a comprehensive overview of the intellectual structure, research hotspots and collaborative patterns in computer vision–based healthcare research. The findings offer valuable insights for researchers, clinicians and policymakers and identify potential directions for future investigations.

## Graphical abstract

Computer vision, powered by machine learning and image processing, plays a transformative role in analysing medical imagery (e.g., MRIs, CT scans, X‐rays), enhancing disease diagnosis, treatment planning and remote patient monitoring. Despite its vast potential, research in this field remains fragmented, limiting comprehensive understanding.

A review of 1023 articles (2000–2022) highlights significant contributors, with IEEE Access as the most active publication venue and China leading in international collaboration. This study provides valuable data on global trends, collaboration networks and future research directions to advance computer vision applications in healthcare.

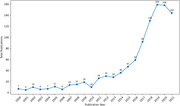

**Abbreviations**
AI: artificial intelligence
AY: active years
CI: citation impact
CNN: convolutional neural network
CU: country affiliations
EnY: ending year
IF: impact factor
MCP: multiple citations
PY: publication year
Q: quartile rankings
SCP: self‐citations
StY: starting year
TC: total citations
TP: total publications
WoS: Wb of science

## 1 Introduction

Computer vision has historically relied on statistical signal processing techniques; however, artificial neural networks and deep learning approaches have rapidly become the dominant methodologies in this field. Recent advances in deep learning have enabled the development of highly accurate algorithms for the classification and interpretation of medical images, including skin lesions and internal anatomical structures [1, 2, 3]. Video‐based data analysis, which contains substantially more information than static diagnostic images such as computed tomography, has shown considerable potential for enhancing clinical decision‐making, particularly in surgical environments. Real‐time video analysis has demonstrated high accuracy in identifying surgical steps and detecting unexpected events during laparoscopic procedures, supporting intraoperative guidance and quality assurance [1, 2].

The integration of computer vision and artificial intelligence (AI) has significantly improved surgical performance, especially in tasks such as suturing and knot tying. Autonomous robotic systems, such as the Smart Tissue Autonomous Robot, have demonstrated superior precision and consistency compared with human surgeons in experimental surgical procedures [1]. Furthermore, algorithms trained on large volumes of surgical video data can recognize anatomical structures and surgical actions, even in complex or emergency scenarios, highlighting the growing role of AI‐assisted systems in modern surgery [2].

Convolutional neural networks (CNNs) are among the most successful deep learning architectures for image recognition tasks. Inspired by the hierarchical processing mechanisms of the human visual cortex, CNNs extract multilevel features from images to enable accurate interpretation of complex visual patterns [3]. Since their early conceptualization, CNN‐based models have shown remarkable success in medical image analysis, including disease detection, classification and prognostic assessment [3]. The availability of large annotated datasets has further accelerated CNN performance through iterative optimization of weights and filters during training [3].

Since the 1950s, AI has progressively influenced healthcare, driven by advances in computational power, data availability and algorithmic innovation [4]. AI technologies now support clinical decision‐making by analysing disease patterns, correlating clinical signs with diagnostic data and assisting healthcare professionals in diagnosis and treatment planning [5, 6]. Numerous studies have demonstrated that AI‐based systems can improve diagnostic accuracy across multiple medical specialties, including dermatology, radiology and ophthalmology [7, 8, 9, 10].

Beyond diagnosis, AI applications have contributed to improved patient management, symptom monitoring and medication adherence. AI‐driven platforms have been shown to enhance treatment compliance and patient engagement, ultimately leading to improved health outcomes [11, 12]. The rapid expansion of AI‐related research has resulted in a substantial increase in scientific publications in healthcare, making it increasingly challenging for researchers and clinicians to remain informed about emerging trends and research priorities.

Bibliometric analysis provides a quantitative and objective approach to evaluating scientific literature by identifying influential authors, institutions, journals and thematic trends within a research domain [13, 14]. Such analyses enable researchers to assess the evolution of a field, detect emerging research hotspots and facilitate interdisciplinary collaboration [14, 15]. Despite the growing body of research on AI in healthcare, bibliometric studies have largely focused on general AI applications or specific disease domains, such as depression and immunotherapy, rather than computer vision–specific applications [13, 16, 17, 18, 19, 20, 21, 22, 23, 24].

The growing application of AI in dentistry further illustrates the expanding scope of computer vision in healthcare. AI‐based systems have been applied to orthodontic diagnosis, treatment planning, robotic‐assisted dental procedures, implant placement and the detection of oral diseases, including caries, periodontal disease, temporomandibular joint disorders and oral potentially malignant lesions [19, 20, 21, 22]. Although several narrative and systematic reviews have explored AI applications in dentistry, comprehensive bibliometric evaluations remain limited [23].

Scientific publication plays a critical role in advancing healthcare research, professional education, and clinical practice. The quantity and quality of published research are increasingly used as indicators of academic productivity and national scientific progress [25, 26]. Bibliometric indicators, such as citation counts, have become essential tools for evaluating research impact at individual, institutional and national levels [27, 28, 29, 30]. These indicators also support the identification of collaborative networks and emerging research themes across disciplines [31, 32].

Modern bibliometric analyses rely on advanced computational tools and machine learning techniques to process large‐scale bibliographic datasets derived from databases such as Web of Science (WoS), Scopus, PubMed and dimensions [27, 28, 29, 30]. Software platforms such as VOSviewer, Bibliometrix and Biblioshiny facilitate visualization of co‐authorship networks, keyword co‐occurrence and citation structures, enabling comprehensive mapping of knowledge domains [33, 34]. Such approaches have been successfully applied to analyse global research trends in diverse scientific areas, including environmental science, public health and human mobility studies [35, 36].

Despite the rapid growth of computer vision research in healthcare, a comprehensive bibliometric analysis focusing specifically on this field remains lacking. Therefore, the present study aims to systematically analyse global research trends in computer vision applications in healthcare using publications indexed in the WoS database from 2000–2022. By examining publication output, citation patterns, collaborative networks, research themes and funding sources, this study seeks to provide a structured overview of the intellectual landscape of medical computer vision research and identify potential directions for future investigation.

## 2 Materials and Methods

A distinct viewpoint can be obtained from bibliometric research when thoroughly analysed. The bibliometrics tool in the R package is designed for quantitative scientometrics and informetrics [37]. Bibliometric technologies make it easier to retrieve information from the repository and classify and analyse vast amounts of historical data gathered from research projects carried out over a given period. In contrast, quantitative techniques are used in bibliometric analysis and meta‐analysis. Unlike systematic literature reviews, which usually rely on qualitative techniques and may be tainted by them, they can prevent or lessen interpretation bias from scholars with different academic backgrounds [38]. This study examined current developments in computer vision‐based research using bibliometric analysis. The methodical, transparent, impartial, and repeatable quantitative statistical evaluation of publications is known as bibliometric analysis. The two primary methods of bibliometric analysis are content analysis and descriptive analysis. The descriptive analysis also includes a review of multiple publications and journal indices to determine how authors and sources can successfully publish their work. Conversely, content analysis makes visible the underlying assumptions of specific academic disciplines. It discovers well‐liked topics, recurrent themes and possible research topics using keyword and citation analyses. Bibliographic data is importable into databases like Scopus, WoS, Dimensions, Lens, Cochrane Library and PubMed. There are distinct goals and features in each of these databases. Almost all fields use the WoS and Scopus as their most popular literature databases [39, 40]. We looked up several publications and more citation‐rich data for this study in the Scopus database [41].

Scholarly publications, patents, clinical trials and policy papers can all be searched and assessed using the integrated database Scopus. We used publication titles, abstracts and keywords to perform an author exploration. The domains covered by this research included genetics and molecular biology, pharmacology, toxicology, immunology and microbiology, medicine, computer science and social sciences. Further question refinement was made possible by the January 2000 to December 2022 publication date range. First, 4323 publications were selected for analysis on 1 December, 2023, when the data was imported. This study included original English‐language articles, book chapters, reviews and conference papers. Reviews, editorials, letters and comments posted on preprint websites were excluded from the study.

To determine which publications exclusively addressed computer vision in healthcare, we reviewed and assessed the published data. With these inclusion and exclusion criteria, we found a set of 1023 scientific papers between January 2000, the earliest accessible date, and December 2022, the latest available date. For the bibliometric analysis conducted for this study, the 1023 records in the dataset served as the basis. A method for searching the WoS database for relevant articles was employed in this study. A CSV file from the WoS database with bibliographic information was downloaded. The Bibliometrix R package was installed after R Studio had loaded. A R console command of biblioshiny() launched Biblioshiny. Non‐programmers can use the Bibliometrix package in R using Biblioshiny, an online tool. To help researchers conduct thorough bibliometric analysis, Bibliometrics offers several tools [23]. By calculating the frequency of keywords that co‐occurred in two scientific articles, the intricate keyword network linkages became simpler to comprehend using the statistical software programme Biblioshiny for data mining in bibliometrics. Excel (.csv) files were uploaded to the Biblioshiny interface only once. To accomplish the study's goals, Excel (.csv) files and portable network graphics (.png) files had to be downloaded to perform data analysis. We extended the scope of computer vision in medical research by extracting additional patterns using VOSviewer. The global publication map was examined using a bibliometrics map viewer and generator called VOSviewer. When you quote a piece of writing, a text‐mining tool can help readers build and visualize a network or relationship. It offers a range of display options and features, such as scrolling, zooming and searching and it can map longer articles and publications.

### 2.1 Data Source

This study's data came from the WoS, a large, reliable and well‐known scientific literature database. The strict inclusion criteria of WoS have led to its reputation for producing high‐quality documents.

### 2.2 Keywords and Data Retrieval

**FIGURE 1:**
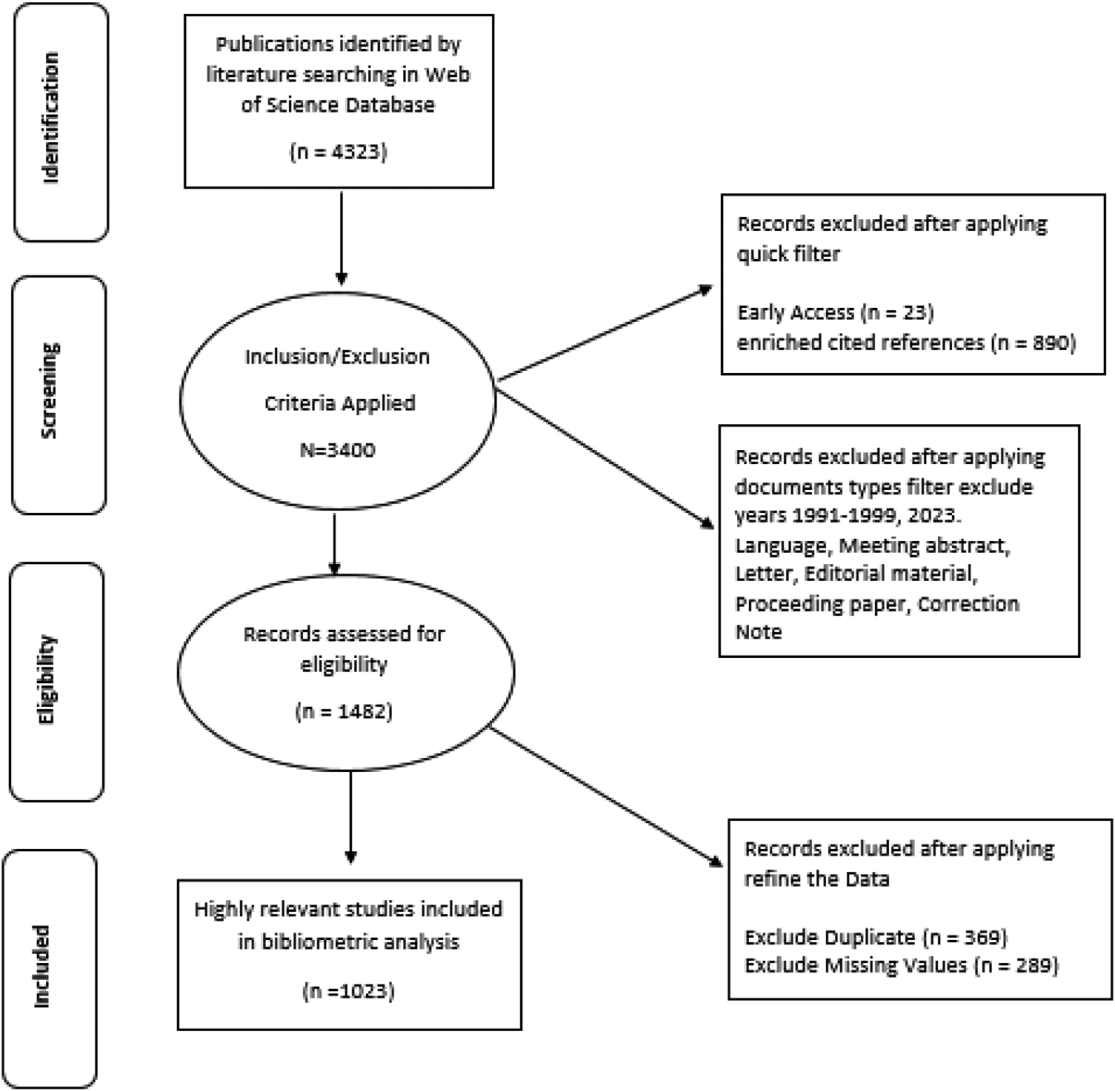
Four‐phase flow diagram for computer vision data extraction and filtering in medical research.

The systematic approach to finding and refining the results is shown in Figure 1. On 1 December, 2023, a literature search was completed to prevent changes in publication numbers and citations brought about by database updates. Two medical scientists working on the project examined relevant literature before developing appropriate search terms for a database search. In order to maximize the number of relevant results, Boolean search operators were added to the keywords. Data retrieval was done using the topic (TS: title, abstract, author keywords and keywords plus) search option of the three editions of the WoS core collection databases: science citation index expanded, social sciences citation index from 1900 to the present and arts and humanities citation index from 2000–2022. It provides more thorough coverage through data searches of the publications' titles, abstracts and keywords. The search formula used for this investigation was:

((TS = (computer vision*) or TS = (machine vision*)) and TS = (medical) and early access (exclude—document types) and enriched cited references and open publisher‐invited reviews and proceeding paper or book chapters or editorial material or data paper or meeting abstract or letter or biographical‐item or meeting or news item or retraction (exclude—document types) and 2024 or 1999 or 1998 or 1997 or 1996 or 1995 or 1994 or 1993 or 1992 or 1991 or 2023 (exclude—publication years (PY)) along with Chinese, German, French, Portuguese, Russian, Spanish, Korean, Italian, Japanese or Turkish (languages not included).

### 2.3 Criteria for Inclusion and Exclusion

Strict inclusion and exclusion standards were implemented to ensure the data was accurate and comprehensive. The WoS was used to search three indexes: the science citation index expanded, the arts and humanities citation index and the social sciences citation index. Only publications that underwent peer review were included in the analysis.

### 2.4 Tools Used

**FIGURE 2:**
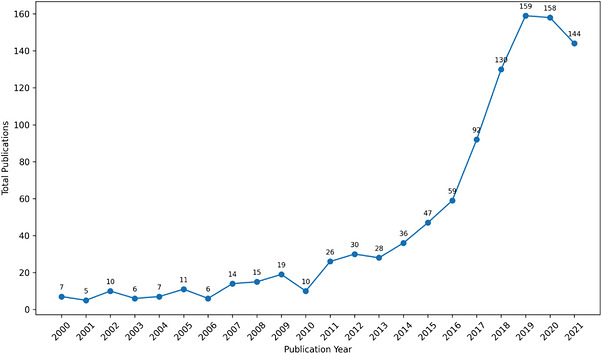
Annual growth of publications related to computer vision applications in healthcare from 2000–2021, showing a marked increase in research output after 2015 and a peak in 2019–2020.

The data was processed, analysed and visualized using VOSviewer, Biblioshiny, ScientoPy, Microsoft Word and Excel as shown in Table 1. • Figures 9, 10, and 11 show the bibliography of authors, journals and countries. • Figure 7 represents the theme evolution, and figure 5 represents citation bursts, and figure 12 represents the three‐field plot. • Figures 6a and 6b show the index keywords and author keywords.• Figure 2 shows the evolution of publications and citations. • Table 6 shows the active authors.

**TABLE 1:** Tools and software used in data processing and visualization.

Tools/software	Description of software	
VOSviewer (version 1.6.19)	VOSviewer can create maps based on text, bibliographic or network data. Furthermore, the application enhances accessibility for both map viewing and exploration.	
Biblioshiny (version 4.1)	The program biblioshiny provides a bibliometrics web interface.	.
ScientoPy (version 2.1.3)	Bibliometric and scientometric analysis software, available as an open‐source project and written in Python.	
Microsoft Excel (2016 version)	Spreadsheets use Microsoft Excel, a powerful Microsoft application, to use functions and formulas to organize and analyse data.	
Microsoft Word (2016 version)	—	Figure 1: Flow diagram

Concerning computer vision research in medicine, the following questions were the focus of this study:

- How much research has been done in medical computer vision and how has it changed?
- What impact has this research had on things?
- Who and how are the collaborators in medical computer vision?
- Which academic institutions, writers and journals are at the forefront of medical computer vision?
- What are the most common keywords and themes in computer vision for medical applications?
- What areas of medical computer vision research are currently being studied?
- According to computer vision in medicine, they have cited references; what relationships exist between writers, journals and countries?
- Which funding agencies support medical computer vision?

## 3 Results

### 3.1 Main Information

The data set includes 1023 documents, spanning 2000–2022 and is derived from 465 sources displayed in Table 2. An astounding 14.73% annual growth rate has been observed during this time. Documents have an average age of 5.46 years, which makes them relatively recent. With an average of 43.36 citations, every document receives excellent attention. References abound, numbering 53,658 in all. The documents cover various topics, as evidenced by 2322 keywords plus and 3844 author's keywords. Collaboration is visible, with 4352 authors and 31.09% of them being international co‐authors. With an average of 4.75 co‐authors per document, minor documents have several authors. The dataset comprises a wide range of research and analysis, as evidenced by the 840 articles and 183 reviews among the document types.

**TABLE 2:** Descriptive statistics and bibliometric characteristics of the analysed dataset (2000–2022).

Description	Results
**Main information about data**	
Timespan	2000:2022
Sources (journals, books, etc.)	465
Documents	1023
Annual growth rate %	14.73
Document average age	5.46
Average citations per doc	43.36
References	53658
**Document contents**	
Keywords plus	2322
Author's keywords	3844
**Authors**	
Authors	4352
Authors of single‐authored docs	35
**Authors collaboration**	
Single‐authored docs	36
Co‐authors per doc	4.75
International co‐authorships %	31.09
**Document types**	
Article	840
Review	183

### 3.2 Annual Productions

Figure 2 shows the total publications (TP) made over a range of PY in a given subject or domain. It has seven publications in 2000 and 144 publications between 2000 and 2022. The trend over time is growing, albeit with sporadic fluctuations. Until 2010, there was a steady increase in publications, but in the years that followed, they increased more quickly, peaking in 2021 with 158 works. Research trends, scientific community interests and advancements may drive the data's indication of a progressive interest and engagement in the field.

### 3.3 Citation Impact

**FIGURE 3:**
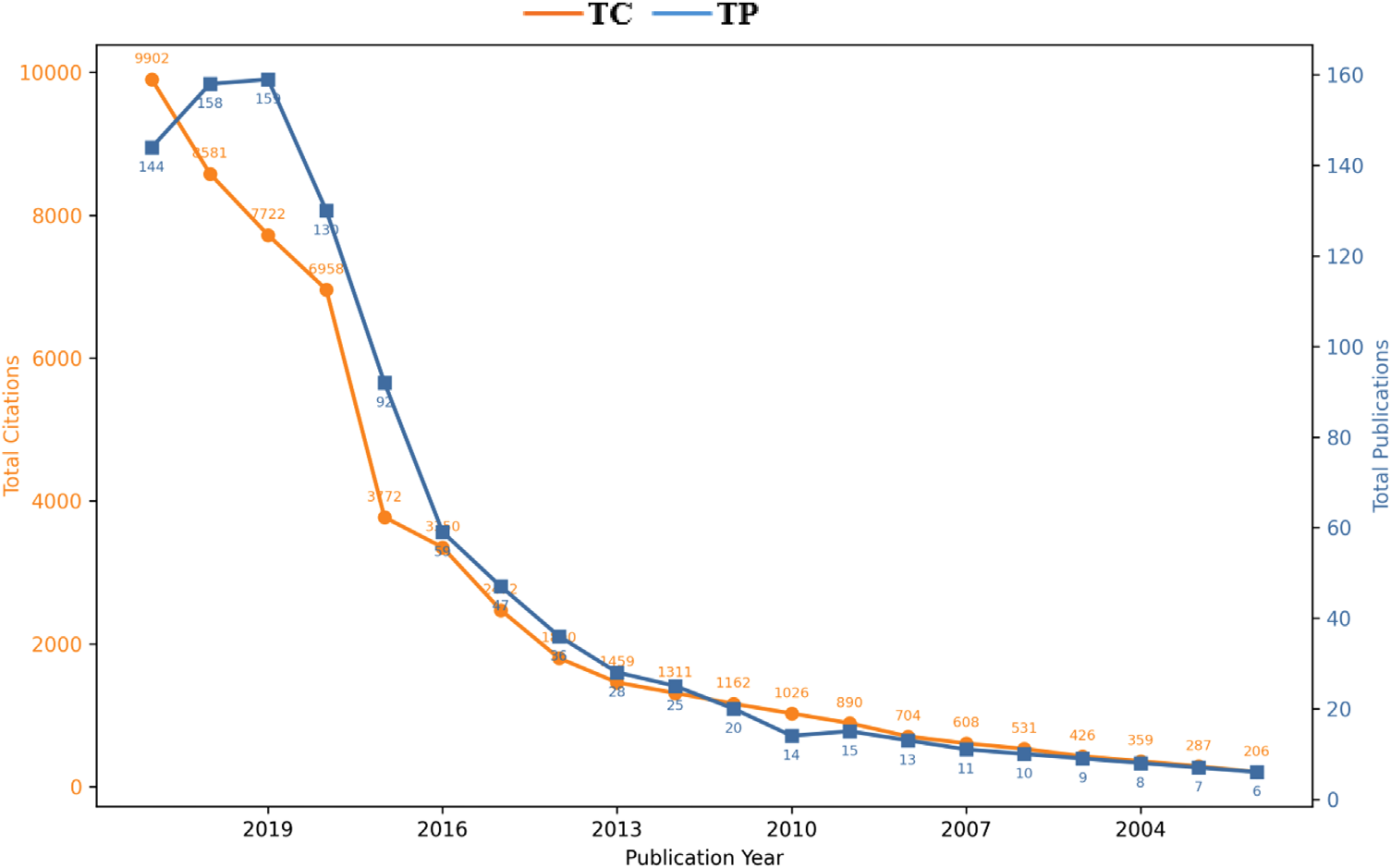
Temporal trends in total publications (TP) and total citations (TC) in computer vision research in healthcare, illustrating publication output and citation impact across years.

Figure 3 presents data on PY, TP and total citations (TC) for a range of years from 2000–2022. Each row indicates a year's worth of publications and citations. For instance, 158 publications in 2021 contained 3772 citations. Significant variations in publication frequency and citation counts are seen when comparing the data from 2001 and 2002 with that from 2019 and 2022. This comparison sheds light on patterns in the output and impact of research over time.

### 3.4 Author Impact

**FIGURE 4:**
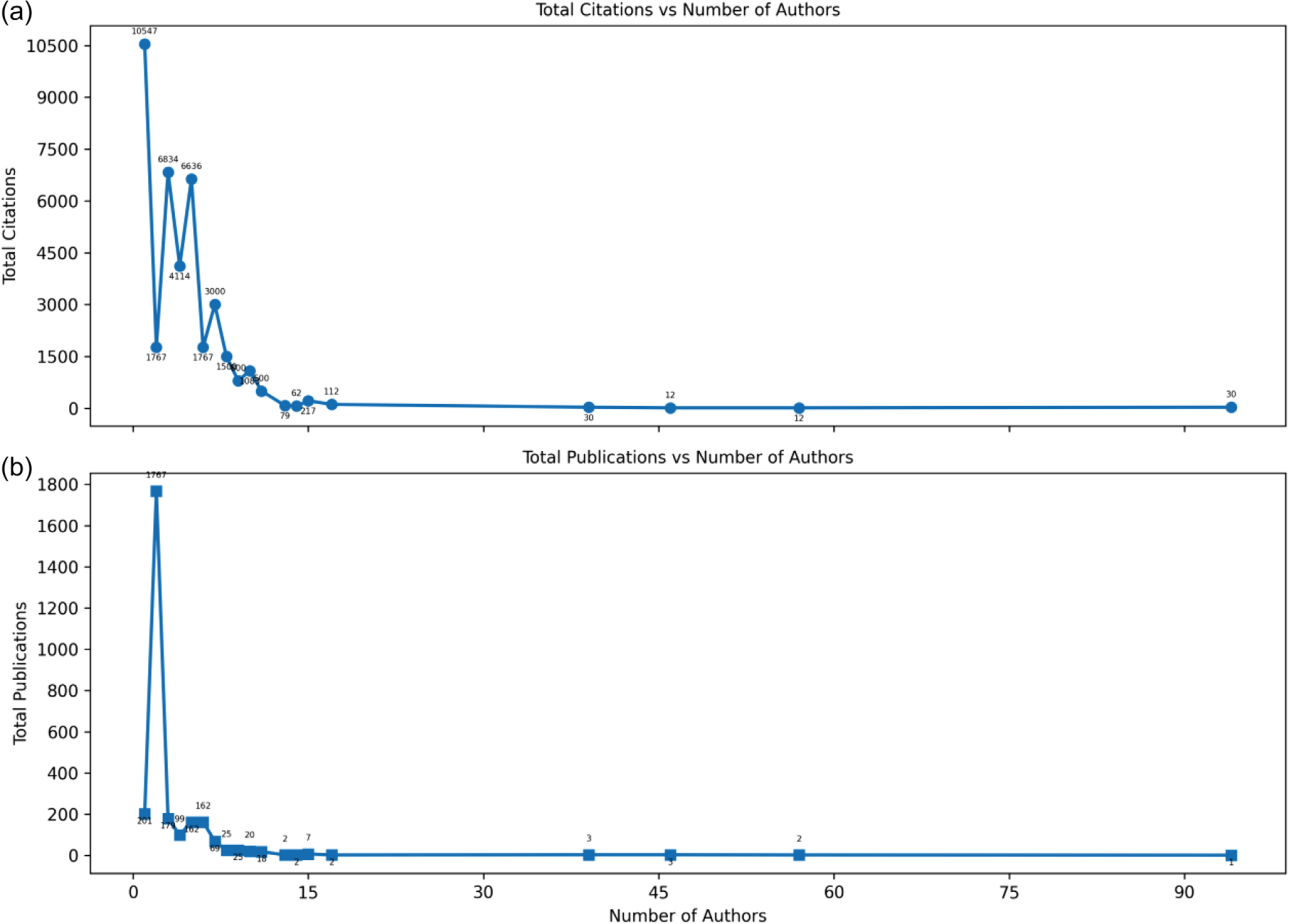
Distribution of (a) total citations (TC); and (b) total publications (TP) according to the number of authors per publication in computer vision research in healthcare.

Figure 4 displays information on the authors' TP and TC. A numerical index identifies each row, which represents an author. Author 3, for instance, has 6834 citations in 179 publications. Likewise, author eight has received 1388 citations from 32 publications. The figure shows that authors differ in their productivity and impact; some have many publications and citations, while others have fewer. Assessing the research output and impact of individual writers within an academic community or field of study is made easier with the help of this information.

### 3.5 Burst Citation

**FIGURE 5:**
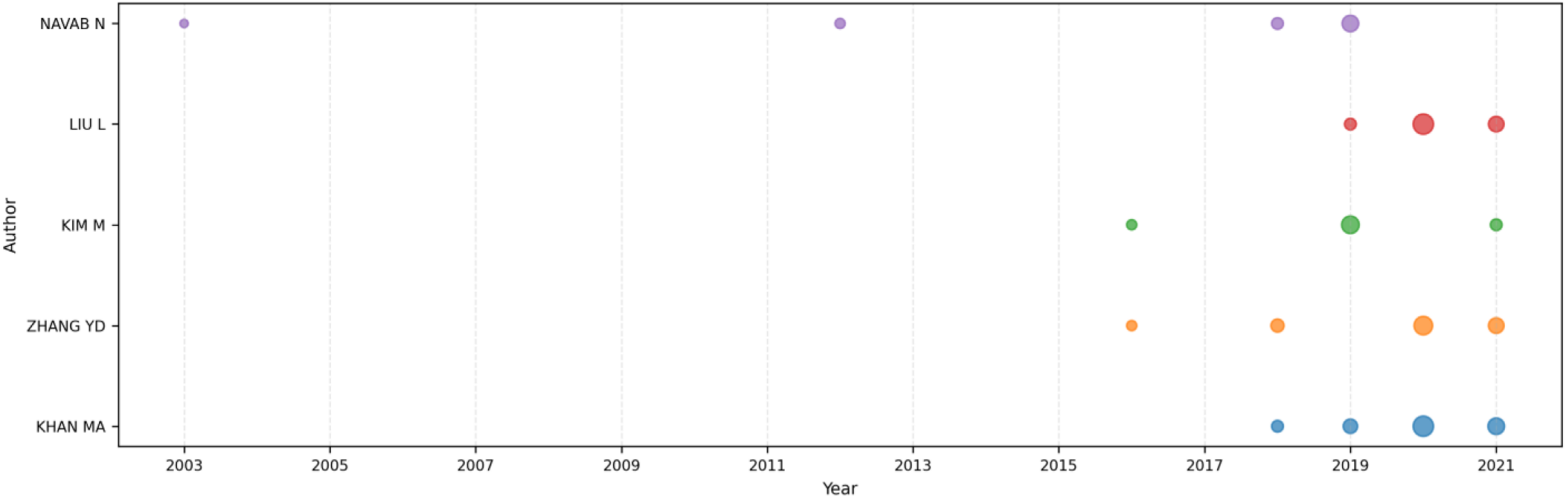
Temporal distribution of publications by the most influential authors in computer vision research in healthcare. Bubble size represents relative publication or citation impact over time.

TP, TC and PY are displayed in Figure 5. With different TP and TC every year, Khan MA, for example, has publications from 2018–2022. Kim M published in 2016, 2019, 2020 and 2022 similarly. With varying TP and TC over time, Liu L's publications span 2019–2022. With notable differences in TP and TC, Navab N's publications from 2003 are included. With a range of TP and TC, Zhang YD's publications cover various topics and exhibit a variable citation rate over three years.

### 3.6 Most Relevant Author, Journal and Affiliations

Table 3 includes several journal titles associated with publishers, place of origin, citation impact (CI), TP, TC, quartile ranking (Q) and impact factor (IF) in the fields of biomedical informatics, robotics and medical imaging. IEEE and Elsevier publish most journals, with most of their publications originating from the US or Europe. Journals like IEEE Transactions on Pattern Analysis, Machine Intelligence and Medical Image Analysis have large TC counts and high CI, demonstrating their influence in their respective fields. Q indicates that a journal stands within the academic community, even though most are listed in the top quartile (Q1).

**TABLE 3:** Top 10 most productive and influential journals in computer vision–Based healthcare research.

Rank	Element	Publishers	CU	TP	TC	CI	Q	IF
1	IEEE access	IEEE	U.S.A.	90	1677	18.63	Q1	4.82
2	IEEE transactions and medical imaging	IEEE	U.S.A.	24	2267	94.45	Q1	11.56
3	IEEE robotics and automation letters	IEEE	U.S.A.	20	368	18.40	Q1	5.89
4	IEEE transactions on pattern analysis and machine intelligence	IEEE	U.S.A.	20	2125	106.25	Q1	21.86
5	Medical image analysis	Elsevier	Netherlands	15	1714	114.26	Q1	12.41
6	Computers in Biology and medicine	Elsevier	U.K	16	493	30.81	Q1	8.88
7	Computer methods and programs in biomedicine	Elsevier	Ireland	18	432	24.00	Q1	7.19
8	IEEE journal of biomedical and health informatics	IEEE	U.S.A.	15	755	50.33	Q1	8.33
9	IEEE transactions on image processing	IEEE	U.S.A.	14	471	33.64	Q1	10.50
10	Pattern recognition	Elsevier	U.K	11	655	59.54	Q1	9.45

*Note*: CU, Corresponding country affiliations; TP, total publications; TC, total citations; CI, citation impact; Q, quartile ranking; and IF, impact factor.

According to various metrics, Table 4 presents a ranking of researchers based on their impact and output within academia. The initials and last name of each researcher identify them. A few of the metrics are active years (AY), starting year (StY), ending year (EnY), TP, TC and CI. Khan MA, for instance, impacts 47 out of 376 citations from eight publications. The AY show how long their academic activity lasted within the specified period. Each researcher's career longevity, productivity and influence over the specified period are all displayed in this ranking.

**TABLE 4:** Top 10 most productive and influential authors in computer vision–based healthcare research.

Rank	Element	TP	TC	CI	StY	EnY	AY
1	Khan MA	8	376	47.00	2018	2022	4
2	Zhang YD	7	409	58.42	2016	2022	6
3	Kumar S	5	119	23.80	2015	2022	7
4	Navab N	6	154	25.66	2012	2020	8
5	Stoyanov D	6	62	10.33	2016	2022	6
6	Acharya UR	5	648	129.60	2014	2022	8
7	Ali H	4	164	41.00	2015	2020	5
8	Ayache N	4	733	183.25	2000	2014	14
9	Damas S	5	161	32.20	2008	2015	7
10	Chua CK	4	116	29.00	2014	2015	1

*Note*: CI, Citation impact; TP, total publications; TC, total citations; EnY, ending year; StY, starting year; and AY, active years.

Table 5 shows the corresponding country affiliations (CU) of several prestigious universities around the world, along with the TP, TC and CI, normalized per paper. Stanford University and Johns Hopkins University are well‐known American universities with a high CI despite having comparatively few publications. Shanghai Jiao Tong University and Zhejiang University are examples of China's robust research output and competitive CI. The University of Washington stands out among American universities because of its significant influence on citations and publication count. The Technical University of Munich best illustrates the extraordinary CI of Germany. The data illustrates the worldwide distribution of research impact and academic quality across various nations.

**TABLE 5:** Leading academic institutions in computer vision healthcare research.

Affiliation	TP	TC	CI	CU
Johns Hopkins University	24	415	17.29	U.S.A.
Shanghai Jiao Tong University	19	724	38.10	China
Stanford University	18	141	7.83	U.S.A.
University of Washington	18	1108	61.55	U.S.A.
Zhejiang University	18	374	20.77	China
Technical University of Munich	16	284	17.75	Germany
University of California	14	1108	79.14	U.S.A.
University of Wisconsin	14	114	8.14	U.S.A.
Chinese University of Hong Kong	13	510	39.23	China
University of Toronto	13	183	14.07	Canada

*Note*: CI, Citation impact; TC, total citations; TP, total publications; and CU, corresponding country affiliations.

### 3.7 Keywords and Thematic Map

**FIGURE 6:**
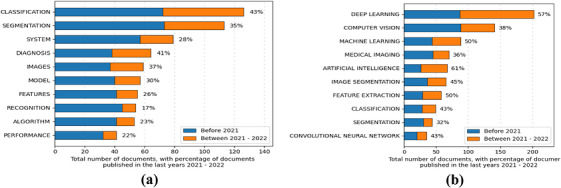
Keywords analysis [index keywords (a), author keywords (b)].

The ranking of keywords in academic literature between 2000 and 2022 is displayed in Figure 6(a). Each keyword has the total number of times it appears in that particular year next to it. ‘Segmentation’ comes in second place with 113 occurrences, and ‘classification’ comes in first place with 126 occurrences. There are additional keywords: ‘diagnosis’, ‘system’ and ‘images’. These keywords′ changing frequency over time indicates shifts in the studied research areas and trends in modeling, algorithm development, diagnosis, classification, segmentation, system development and image analysis.

The author keywords are ranked in Figure 6(b) according to their frequency in scholarly publications between 2000 and 2022. Following years of steady popularity, ‘deep learning’ became the most popular keyword. There is a noticeable interest in ‘computer vision’ and ‘machine learning’ after that. Important research topics like ‘medical imaging’ and ‘AI’ are also highlighted. Moreover, terms like ‘image segmentation,’ ‘feature extraction’ and ‘classification’ refer to the fundamental techniques used in these domains. The term ‘CNN’ highlights the growing focus on image‐processing tasks and deep‐learning architectures.

**FIGURE 7:**
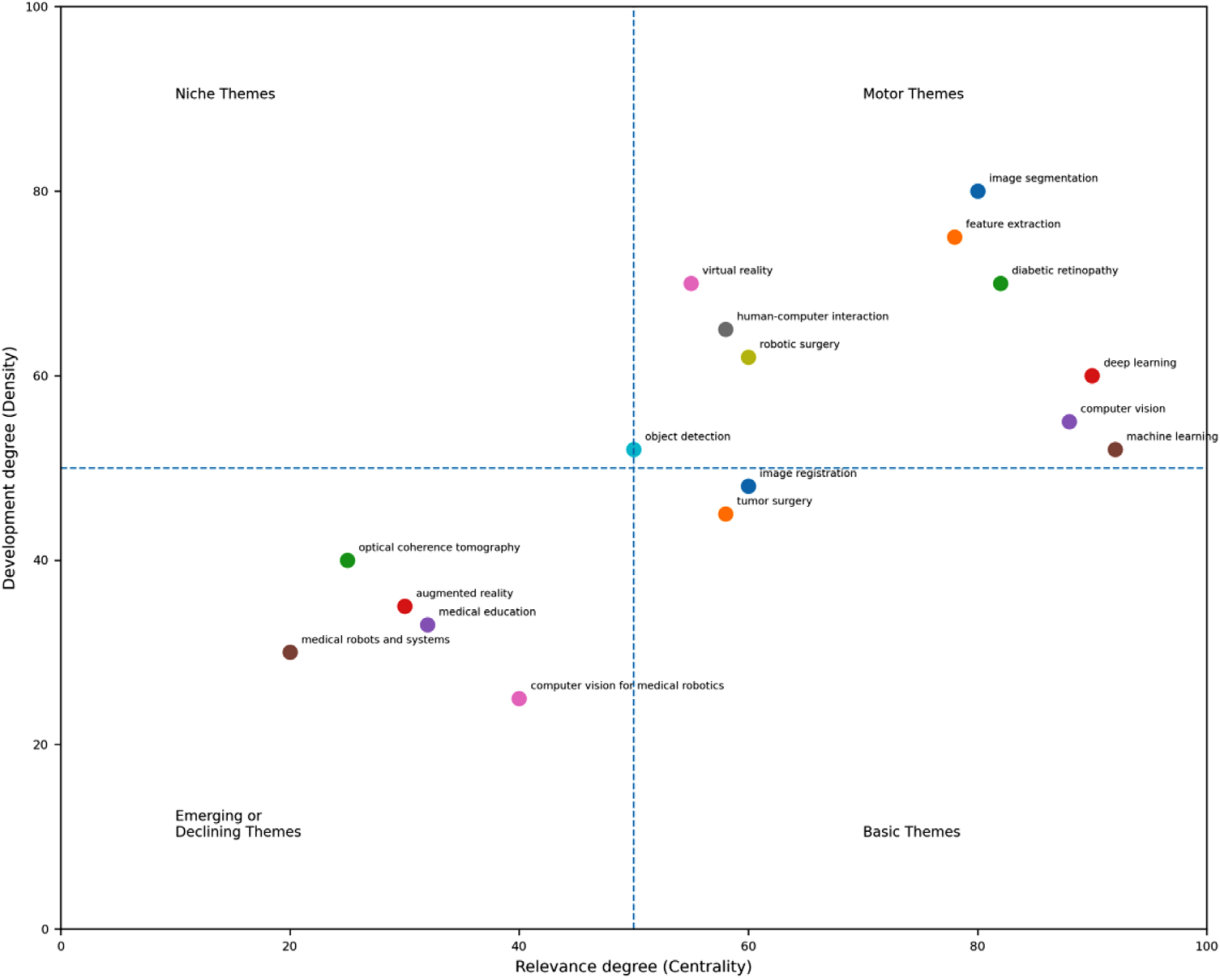
Thematic map illustrating the evolution of research themes in computer vision–based healthcare studies based on centrality (relevance) and density (development). Themes are categorized into motor, basic, niche and emerging or declining clusters.

Keywords can be grouped into themes (i.e., single circles and two‐dimensional images) according to density and centrality. The thematic map as shown in Figure 7 arranges themes into quadrants based on their location; fundamental themes are located in the lower‐right quadrant, while motor themes are located in the upper‐right quadrant. The upper‐left quadrant contains highly specialized/niche topics, while the lower‐left quadrant is occupied by themes that are either emerging or disappearing. The phrase ‘theme evolution’ describes the different evolutionary relationships that represents the development point of the thematic substance, evolutionary pathways and evolutionary drifts, as well as the growth of the field. Finding and identifying relevant topics—like theme shifts and advances in computer vision in healthcare research—was the primary objective between 2000 and 2022. Phrases like computer vision for medical robotics and optical coherence tomography denote continued research in specific medical applications.

### 3.8 Research Area

Table 6 shows that computer vision, a subset of computer science, is the most popular research area, with 205 records, or 20.04% of the total. With 174 entries (17.01%) and engineering trails in the field, this field seeks to empower machines to analyse and comprehend visual data from the real world. It frequently collaborates with computer vision to develop hardware and image processing systems. Nuclear medicine, radiology and medical imaging research also substantially influence the development of computer vision, especially in medical image analysis, with 65 records (6.35%). These graphs show how computer vision has a broad and profound impact on many fields, encouraging innovation in technology and healthcare.

**TABLE 6:** Distribution of publications across research areas in computer vision–based healthcare studies.

Research areas	Record count	% of 1023
Computer science	205	20.03
Engineering	174	17.00
Radiology nuclear medicine medical imaging	65	6.35
Telecommunications	62	6.06
Medical informatics	46	4.49
Mathematical computational Biology	36	3.51
Imaging science photographic technology	33	3.22
Mathematics	29	2.83
Robotics	28	2.73
Science technology	25	2.44

### 3.9 Funding Agencies

**FIGURE 8:**
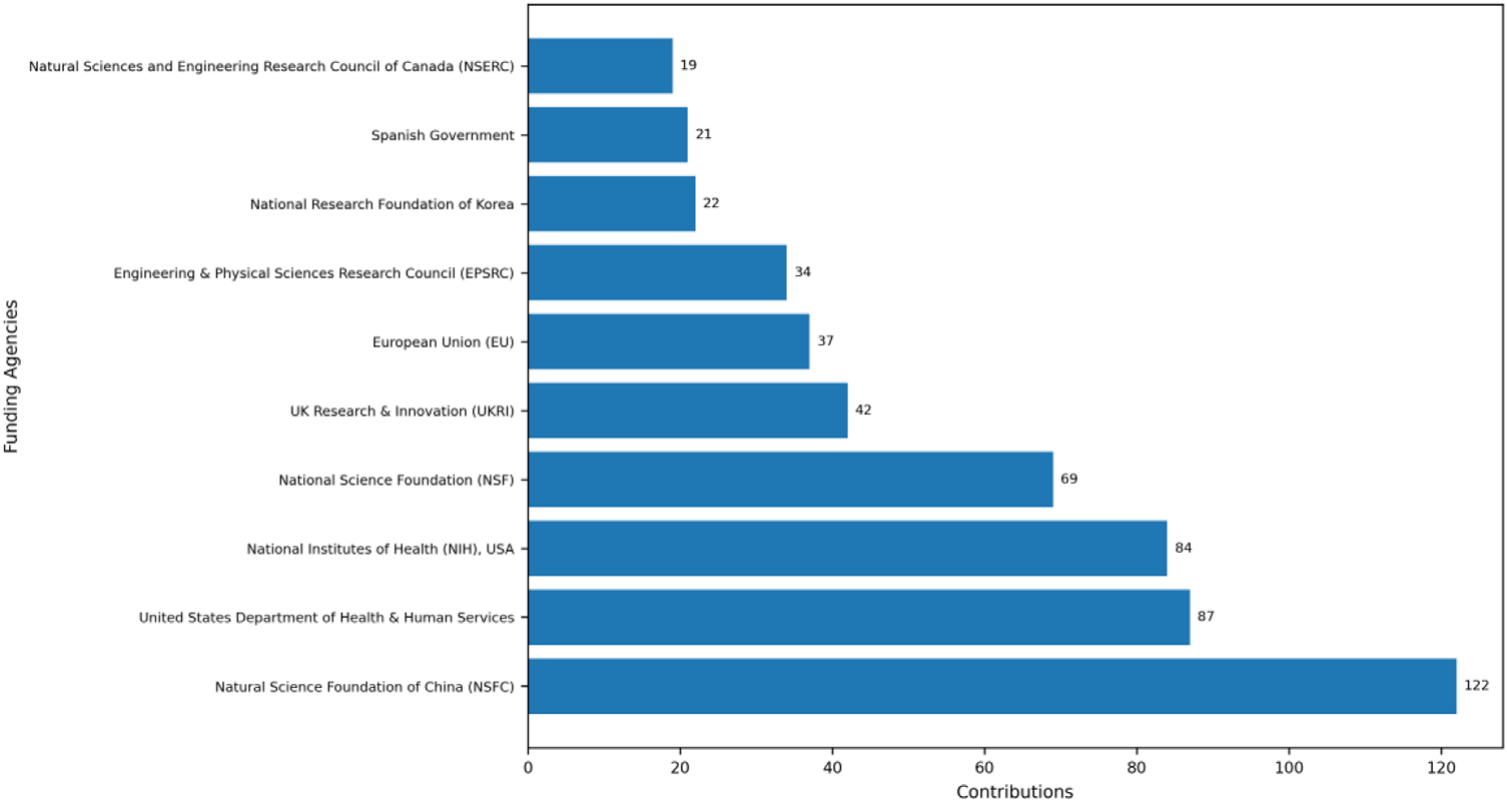
Major funding agencies supporting computer vision research in healthcare, ranked according to the number of contributions.

A global investment in computer vision research is shown in Figure 8, which displays funding agency contributions. With an impressive 122, the United States Department of Health and Human Services and the National Institutes of Health rank second and third, respectively, demonstrating a solid dedication to advancing computer vision applications in healthcare. At the top of the list is the National Natural Science Foundation of China. Other notable donors include the National Science Foundation in the U.S.A., UK Research and Innovation and the European Union. These investments serve as a reminder of the importance of computer vision to numerous global scientific and industrial initiatives.

### 3.10 Bibliography Coupling Sources, Organizations and Countries

**FIGURE 9:**
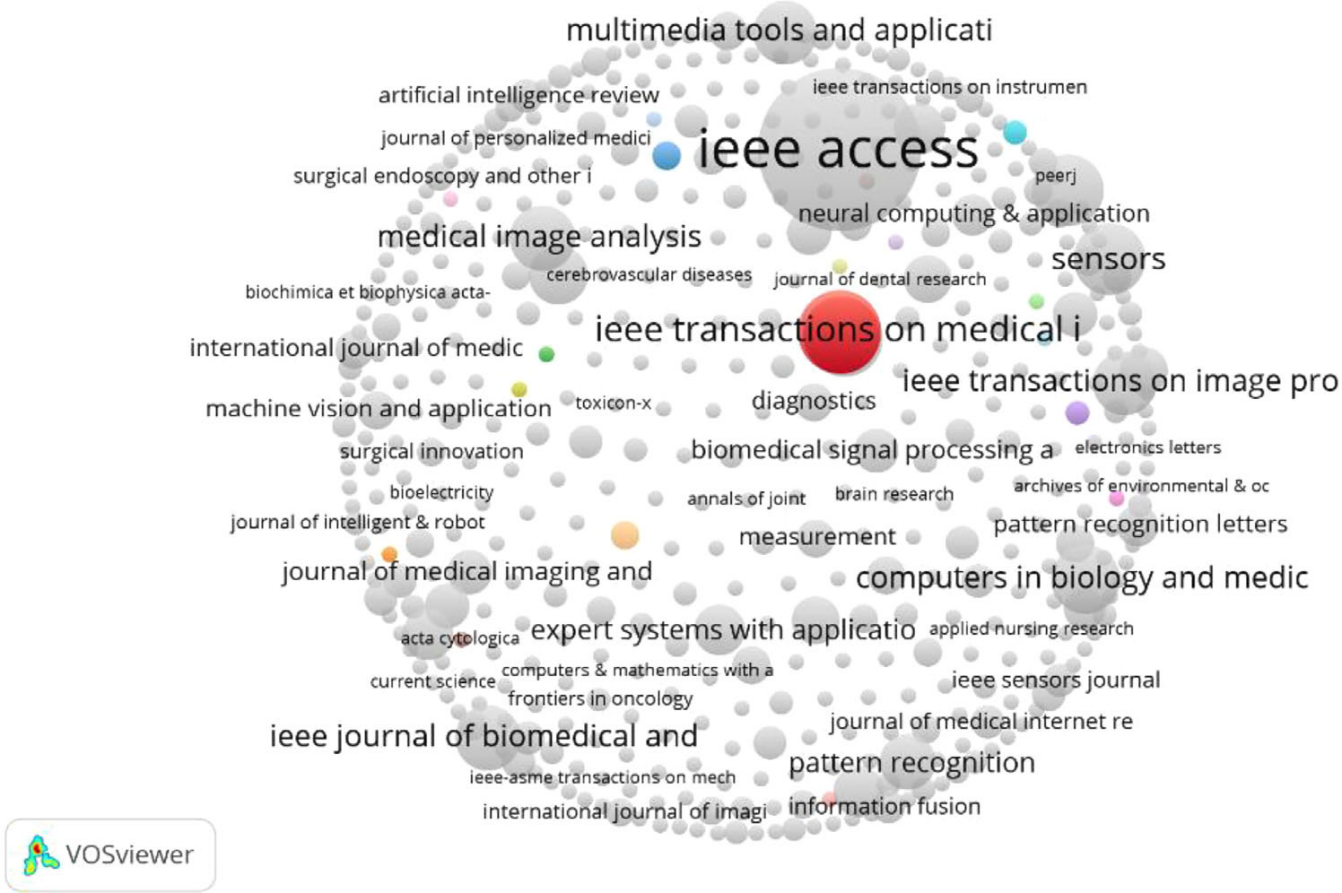
Journal co‐citation network in computer vision–based healthcare research, highlighting the most influential publication sources and their citation relationships.

The number of source documents and related citations from different academic journals is shown in Figure 9 through a network map visualization. Of them, IEEE Access has the most citations (1677) and source documents (90), followed by IEEE transactions on medical imaging (24 documents, 2267 citations). Notable for its noteworthy contribution is IEEE transactions on pattern analysis and machine intelligence, which has 20 papers and 2125 citations. Regarding source documents and citations, other journals like medical image analysis, neurocomputing and information fusion make a modest contribution. In contrast, journals with fewer documents and citations are insights into imaging and journal of vision.

**FIGURE 10:**
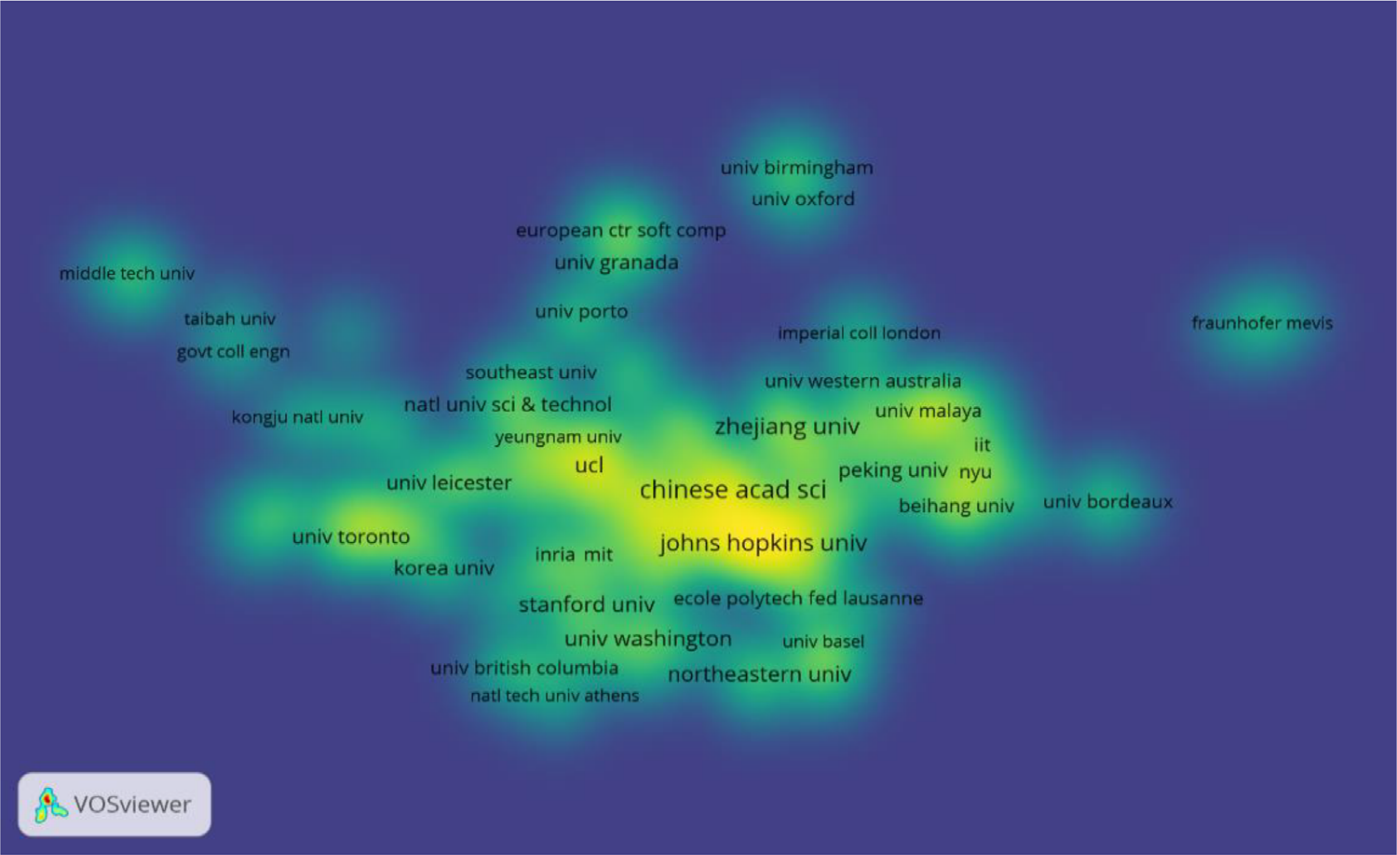
Density visualization map of institutional collaboration in computer vision–based healthcare research.

Figure 10 uses a density visualization map to show the frequency of document citations for different academic institutions and their prominence in scholarly works. The University of Zhejiang, which has had 17 papers mentioned 1018 times, is in first place, with Johns Hopkins coming in second with 14 papers cited 415 and 374 times, respectively. A few other prestigious universities are Shanghai Jiao Tong University, Arizona State University and Technical University Munich. These measurements show the scope of academic output and impact, highlighting the importance of these organizations in furthering scholarly discourse across various.

**FIGURE 11:**
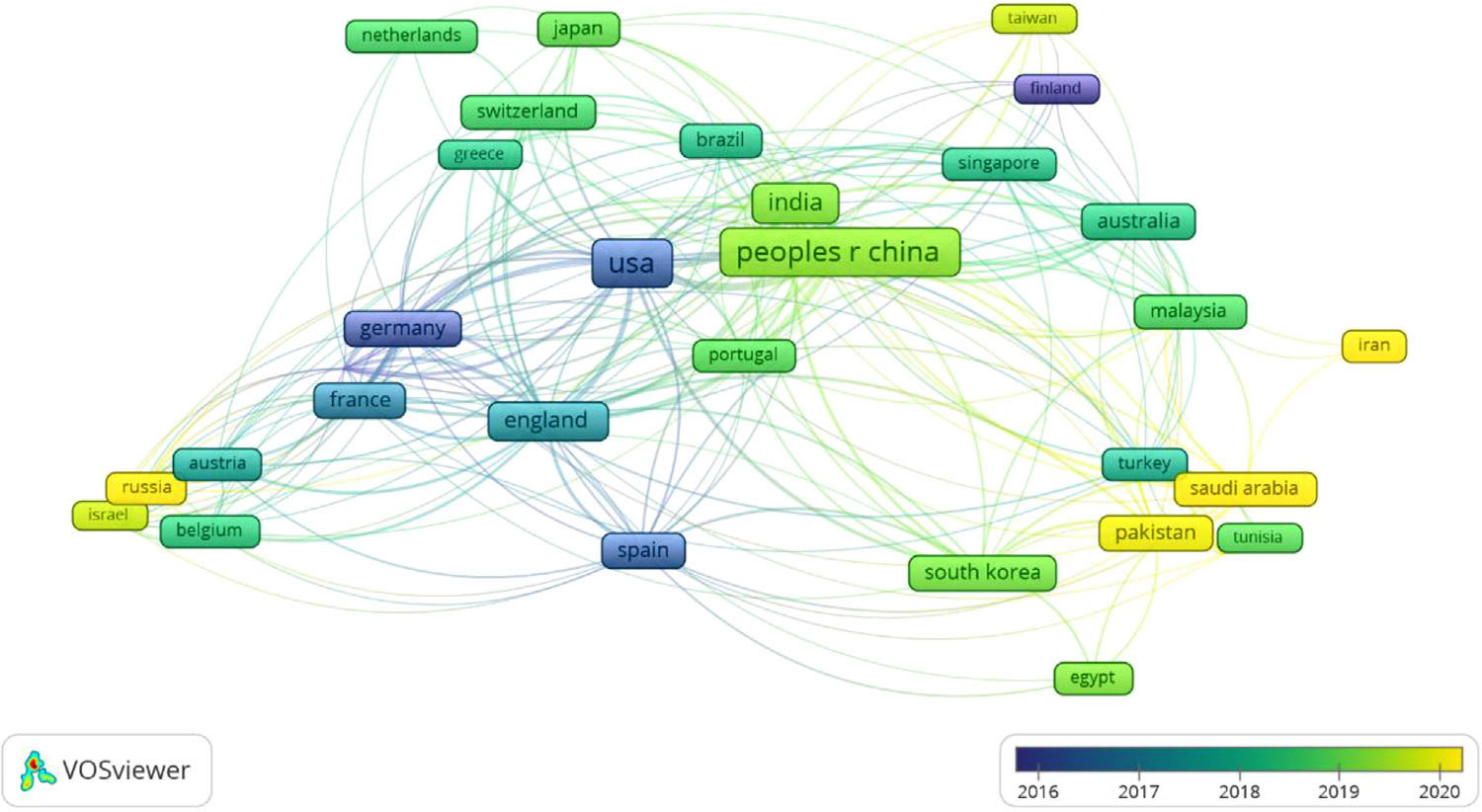
International collaboration network of countries contributing to computer vision research in healthcare.

Figure 11 provides an overview of the scientific productivity of different countries based on the number of publications published and the corresponding citation counts using an overlay visualization map. China tops the list with 171 documents and 2610 citations, followed by Taiwan, Australia and France. Finland is noteworthy for having a high research impact despite having fewer publications and many citations. Nations like Austria, Japan and Iran also display notable research output. Certain countries, like Saudi Arabia and India, exhibit lower citation counts than the total number of documents, which may indicate areas where research visibility or quality could be enhanced. The U.S.A. leads the world in documents, but its citation count is lower, possibly because of the number of publications produced.

## 4 Discussion

This bibliometric analysis provides a comprehensive overview of global research trends in computer vision applications in healthcare from 2000–2022. The findings demonstrate a substantial and consistent growth in scientific output, particularly after 2018, reflecting the increasing integration of deep learning and image analysis techniques into medical research. China and the U.S.A. emerged as the most influential contributors in terms of publication volume, CI and international collaboration.

The dominance of journals such as IEEE access and IEEE transactions on medical imaging highlights the interdisciplinary nature of this research area, bridging computer science, engineering and clinical medicine. Keyword analysis revealed that deep learning, image segmentation and classification remain central research themes, indicating a strong focus on automated diagnostic and decision‐support systems. These findings align with previous bibliometric studies examining AI‐driven healthcare research, confirming the rapid maturation of computer vision technologies in medical applications [18, 19, 20, 21, 22].

**FIGURE 12:**
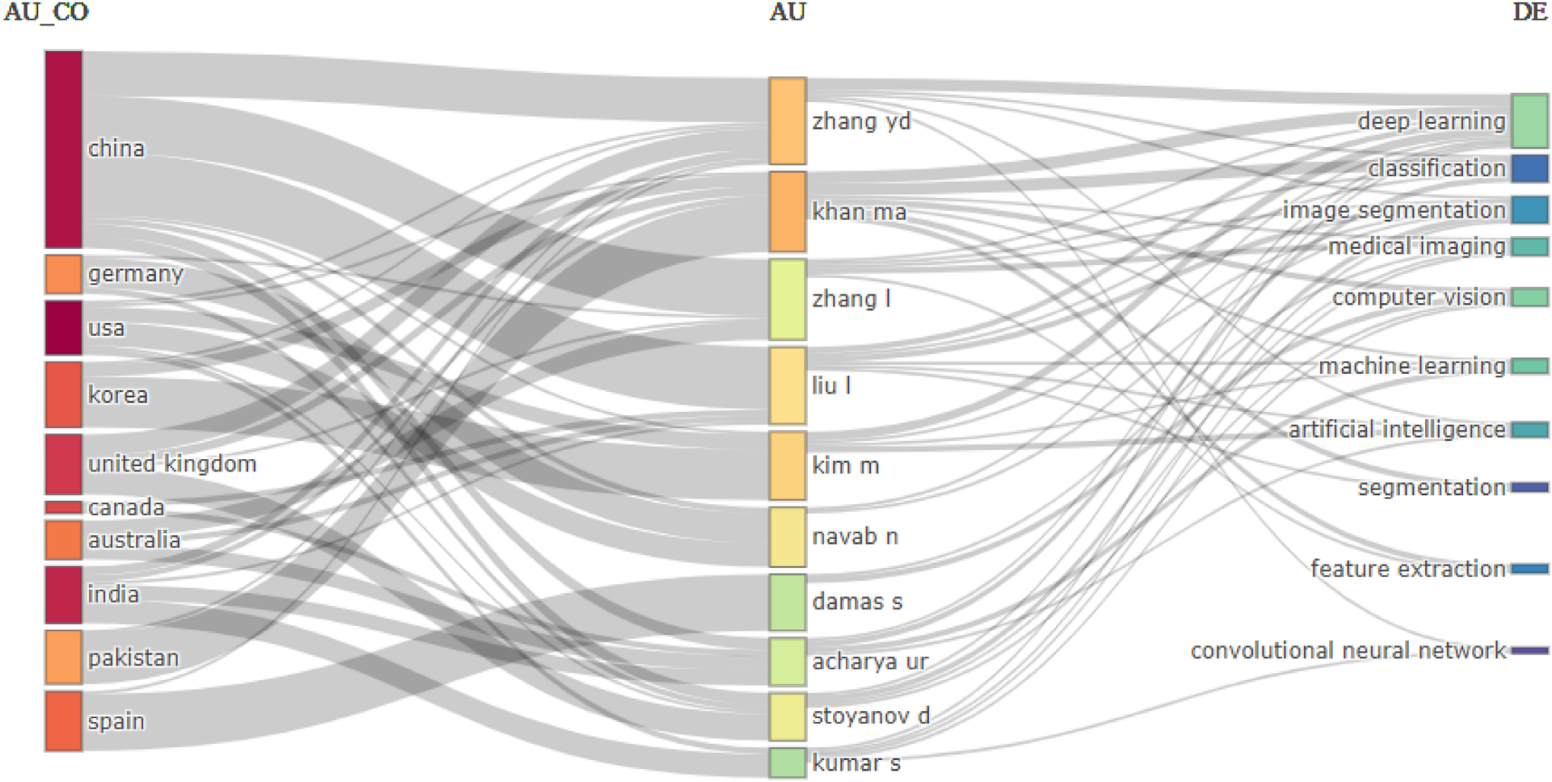
Sankey diagram illustrating the relationship between contributing countries, prolific authors and dominant research themes in computer vision–based healthcare research.

Regarding academic citation metrics, China ranks second with 5899 citations, while the United States leads the pack with an impressive 13,636. Switzerland, Australia and India have also made significant contributions; their total citation counts are 2543, 2094 and 3050, respectively. With citation counts ranging from 1858–1,549, France, Japan and the UK closely follow in order of precedence. With 1446 and 1091 citations, respectively, Canada and Pakistan round out the list. In terms of citation types, the US has the greatest TC score (181), which is made up of 145 multiple citations (MCP) and 36 self‐citations (SCP). China closely trails the United States with a 177, TC driven mainly by 123 SCP and 54 MCP. TC of 95 indicates that SCP (78) is significantly more prevalent than MCP (17) in India. The information presented here shows the different degrees of international research engagement and influence by drawing a line through research output and collaboration patterns across national boundaries. The Sankey plot, a three‐field diagram, clarifies the relationships between countries, writers and keywords in computer vision in medical research. Plotting's vertical dimension shows the cumulative relationships between its elements, with each relevant element represented by a different coloured rectangle. Rectangles that are raised indicate the elements that have the highest relative values. Saneky diagrams illustrating national and author productivity are shown in Figure 12, along with the main contributions of each to computer vision research in medicine. Prominent writers from China, Germany and the U.S.A. stand out as innovators when investigating essential research subjects. The density of links between different sets of values indicates the amount of information flow between them and the Sankey diagram graphically conveys the distribution of entities (countries, authors and keywords).

Research on computer vision in healthcare and related fields saw a sharp uptick in the first quarter of 2020, with publications on the subject increasing dramatically since 2018. Researchers worldwide have researched diagnosis, treatment, management and prevention in healthcare settings. This corpus contains a variety of academic formats, such as meta‐analyses, systematic reviews and research articles. Very little focus has been placed on bibliometric scrutiny per se, even though bibliometric analyses play a crucial role in explaining research trends, volume and interactions between medical practitioners and academics.

According to bibliometric analyses, a robust collaborative culture is evident in this field, as demonstrated by the 36 documents that a single author contributed, although most publications are co‐authored. Sankey diagrams, which utilize three main metadata parameters, provide insightful explanations of domain relationships, author associations with particular keywords and national contributions to this research project. Prominent researchers like Zhang YD and Khan MA have significantly impacted the direction of computer vision research in the medical field, with noticeable increases in output seen after 2008.

Acknowledging the inherently interdisciplinary character of computer vision research, this paper provides an extensive synopsis of current developments and future paths. Computer vision application in healthcare has been the subject of many bibliometric studies, but the field's evolutionary trajectory presents a challenging environment for scholarly research. To determine notable authors, significant references, institutional affiliations, national contributions and disciplinary foci, this study performs a bibliometric analysis of computer vision publications in the healthcare industry using the WoS database.

The findings show that, despite a growing body of research being acknowledged due to publications' widespread accessibility, a small number of writers dominate in creating innovative works. The rising citation rates demonstrate how important this field is becoming in today's conversation. The multidisciplinary nature of the research, as evidenced by the diverse range of methodologies and thematic specializations among eminent scholars, is the primary advantage of studies in this field over those that solely concentrate on systematic reviews. This study is the first bibliometric analysis of the literature from 2000–2022 on computer vision in healthcare. An assessment of the quality of publishers and publications is included to improve the study's analytical rigor.

### 4.1 Limitations

This review had several limitations. It relied solely on the WoS database, which may have excluded relevant studies from other sources, potentially narrowing the scope. Publication bias was also a concern, as the focus on high‐impact journals may have overrepresented certain regions or research types while overlooking emerging or less mainstream studies. Additionally, the temporal range of 2000–2022 may not have captured the most recent developments in rapidly evolving fields like AI and computer vision, indicating the need for future updates using broader databases and more current data.

## 5 Conclusion

Our study attempts to provide an extensive summary of the latest results in medical computer vision research. Utilizing bibliometric analysis, we investigated various aspects of this topic. To be more precise, we identified noteworthy articles, authors, and organizations by combining bibliographic coupling with network and co‐occurrence analyses. With this method, we thoroughly understood the state of this field's research. Furthermore, we utilized bibliographic coupling analysis to identify recurrent themes within the corpus of prior research, providing insight into the study's overall objectives and theoretical frameworks. By offering important new insights with implications for theoretical frameworks and practical applications, our research contributes to the body of knowledge. In addition, our study poses significant theoretical queries regarding subsequent research on computer vision technology in the medical field. Our results allow the exploration of previously unexplored areas by clearly defining research paths in this field, highlighting the current state of play and drawing boundaries for interested scholars. Additionally, our analysis identifies important industry participants who might be engaged in joint ventures and research initiatives. Bibliographic coupling facilitates the discovery of foundational works and generates thematic clusters that offer valuable insights for future researchers. Our study advances methodological rigor in research endeavors through the synthesis of mathematical modelling and empirical analysis, offering a solid foundation for future research on computer vision technology in the healthcare sector.

## Author Contributions

R.I. and H.M.Z. implemented the research, collected the data and drafted the manuscript. A.I. drafted the manuscript and designed the tables. S.A., S.I., and N.P. reviewed the paper for important intellectual content. M.N.B. approved the final version of paper to be published. All authors discussed the results and commented on the manuscript.

## Funding

The authors have nothing to report.

## Conflicts of Interest

The authors declare no conflicts of interest.

## Data Availability

The data set used in the current study will be made available on request from the corresponding authors.
